# Enhancing Clinical Trial Sites in Low- and Middle-Income Countries to Facilitate Product Development in Response to the COVID-19 Pandemic

**DOI:** 10.1093/cid/ciaf094

**Published:** 2025-07-22

**Authors:** Sophie S Y Kang, Birkneh Tilahun Tadesse, Hyon Jin Jeon, Mosoka P Fallah, Nebiyu Dereje, Olayinka Stephen Ilesanmi, Solomon Mequanente Abay, Betelhem I Tekle, Hayoung G Son, Laxman Shrestha, Anita Shrestha, Hasia V Ramiso, Vincent Canouet, Yeonseon Kim, Kwaku Poku Asante, Japhet Anim, Seyram Kaali, Maria Rosario Capeding, Ilesh V Jani, Igor Capitine, Arlete Mahumane, T Anh Wartel, Andrea Haekyung Haselbeck, Tarun Saluja, Florian Marks

**Affiliations:** International Vaccine Institute, Seoul, Republic of Korea; Centre for Tropical Medicine and Global Health, Nuffield Department of Clinical Medicine, University of Oxford, Oxford, United Kingdom; International Vaccine Institute, Seoul, Republic of Korea; Department of Global Public Health, Karolinska Institute, Stockholm, Sweden; Center for Innovative Drug Development and Therapeutic Trials for Africa, College of Health Sciences, Addis Ababa University, Addis Ababa, Ethiopia; Heidelberg Institute of Global Health, University of Heidelberg, Heidelberg, Germany; International Vaccine Institute, Seoul, Republic of Korea; Cambridge Institute of Therapeutic Immunology and Infectious Disease, University of Cambridge School of Clinical Medicine, Cambridge, United Kingdom; Madagascar Institute for Vaccine Research, University of Antananarivo, Antananarivo, Madagascar; Africa Centres for Disease Control and Prevention, Science and Innovation Directorate, Addis Ababa, Ethiopia; Africa Centres for Disease Control and Prevention, Science and Innovation Directorate, Addis Ababa, Ethiopia; Africa Centres for Disease Control and Prevention, Science and Innovation Directorate, Addis Ababa, Ethiopia; International Vaccine Institute, Seoul, Republic of Korea; Department of Pharmacology and Clinical Pharmacy, Addis Ababa University, Addis Ababa, Ethiopia; International Vaccine Institute, Seoul, Republic of Korea; International Vaccine Institute, Seoul, Republic of Korea; Institute of Medicine, Tribhuvan University Teaching Hospital, Kathmandu, Nepal; Institute of Medicine, Tribhuvan University Teaching Hospital, Kathmandu, Nepal; Tropical Disease Foundation, Makati City, Philippines; International Vaccine Institute, Seoul, Republic of Korea; International Vaccine Institute, Seoul, Republic of Korea; Cambridge Institute of Therapeutic Immunology and Infectious Disease, University of Cambridge School of Clinical Medicine, Cambridge, United Kingdom; Kintampo Health Research Centre, Research and Development Division, Ghana Health Service, Kintampo, Ghana; Kintampo Health Research Centre, Research and Development Division, Ghana Health Service, Kintampo, Ghana; Kintampo Health Research Centre, Research and Development Division, Ghana Health Service, Kintampo, Ghana; Tropical Disease Foundation, Makati City, Philippines; Asian Hospital and Medical Center, Muntinlupa City, Metro Manila, Philippines; Instituto Nacional de Saùde, Maputo, Mozambique; Instituto Nacional de Saùde, Maputo, Mozambique; Instituto Nacional de Saùde, Maputo, Mozambique; International Vaccine Institute, Seoul, Republic of Korea; International Vaccine Institute, Seoul, Republic of Korea; International Vaccine Institute, Seoul, Republic of Korea; International Vaccine Institute, Seoul, Republic of Korea; Heidelberg Institute of Global Health, University of Heidelberg, Heidelberg, Germany; Cambridge Institute of Therapeutic Immunology and Infectious Disease, University of Cambridge School of Clinical Medicine, Cambridge, United Kingdom; Madagascar Institute for Vaccine Research, University of Antananarivo, Antananarivo, Madagascar; The Hong Kong Jockey Club Global Health Institute, Hong Kong Special Administrative Region, China

**Keywords:** vaccine, COVID-19, clinical trial, capacity enhancement, pandemic preparedness

## Abstract

**Background:**

The swift development of coronavirus disease 2019 (COVID-19) vaccines marked a monumental effort in global coordination and collaboration; however, there remained major disparities in vaccine access and research capacity across countries. Unequal participation in vaccine development studies from low- and middle- income countries (LMICs) clearly signaled an urgent need to strengthen health research infrastructure in those regions.

**Methods:**

With funding from the Gates Foundation (GF), this site readiness initiative carried out rapid capacity enhancement activities to enable large-scale, Phase 3 pivotal clinical trial conduct in LMICs. The International Vaccine Institute (IVI) worked with site partners in four countries (Mozambique, Ghana, Nepal, and the Philippines) after conducting feasibility assessments for site selection. Site-specific gaps were identified, and capacity building activities focused on staff training, site infrastructure, and resource mobilization were carried out over roughly 7 months from October 2020 to May 2021.

**Results:**

Despite pandemic-related challenges such as supply chain shortages, by the end of the capacity building efforts all sites were either contracted to or in discussions with trial sponsors to conduct severe acute respiratory syndrome coronavirus 2 (SARS-CoV-2) vaccine studies. This article provides an overview of the site selection process, critical components of site establishment, and final site readiness evaluations carried out amidst a global health emergency.

**Conclusions:**

This experience illustrates the value of research capacity enhancement as essential to both pandemic preparedness and global health equity. The lessons learned are being carried into an ongoing initiative across West Africa, currently underway as the “Advancing Research Capabilities in West Africa (ARC-WA).”

Decades prior to the coronavirus disease 2019 (COVID-19) pandemic, the World Health Organization (WHO) identified the need to prioritize health research capacity advancement in countries with the greatest vulnerabilities to health emergencies, and emphasized that strengthening these systems during inter-epidemic periods was vital [1]. However, sustainability is a challenge in this regard, requiring short- and long-term commitment and an integrated approach that targets individuals, institutions, and countries to establish robust health research systems [2]. A disparate burden has been imposed on low- and middle-income countries (LMICs) compared to high-income countries (HICs) in implementing such infrastructure; the gaps left by the failure to establish a sustainable health research system and equal capacity advancement across all countries compromises global health security.

These gaps and their detrimental consequences were made evident during the COVID-19 pandemic. The global effort toward developing vaccines was unparalleled in scale and speed, and the response showcased the possibility of impressive collective and cooperative action towards a common goal [3, 4]. Nonetheless, COVID-19 exposed the challenges of developing vaccines globally, leading to marked differences in countries’ ability to cope with the pandemic [5, 6]. A 2021 meta-analysis demonstrated that LMICs experienced a higher burden of COVID-19 compared to HICs, primarily due to vaccine inequity [7].

In particular, the pandemic highlighted the inadequate worldwide geographical representation of vaccine clinical trials [4]. During early development of SARS-CoV-2 vaccine candidates, clinical trials were disproportionately concentrated in Europe and North America, with much more limited representation from Africa, South and Southeast Asia, Central America, and South America [4]. Approximately 81% of trials were conducted in high- and upper-middle income countries and 19% conducted in low- and lower-middle income countries [8]. These disparities between HICs and LMICs necessitate a call to action on vaccine and research equity for global security—underscoring the need for a global health architecture such as the Pandemic Agreement that ensures inclusivity and equity in accessing essential countermeasures during public health emergencies [9].

Cultivating research capabilities within LMICs is essential to conducting rigorous, locally led, and context-specific studies that can inform effective infectious disease control strategies, and requires substantial support and leadership [10]. The African Centers for Disease Control (Africa CDC) and its African partner countries, with the support from major donors such as the Coalition for Pandemic Preparedness and Innovation (CEPI), have launched a vaccine development initiative to support manufacturing, clinical trials, and the widespread deployment of novel vaccines in Africa [11]. Notably, the African Union has targeted local manufacturing of 60% of vaccines required in Africa by 2040 [12]. Meeting this goal will require establishing robust infrastructure to test, regulate, manufacture, and distribute medical products [13].

A key part of vaccine development is the collection and evaluation of safety and efficacy data within defined regulatory frameworks. Performing these evaluations under time pressure during public health emergencies presents methodological and operational challenges [4]. As seen early in the COVID-19 pandemic, strong clinical trial sites were in high demand to swiftly commence efficacy trials once vaccine candidates emerged from Phase 2 trials. In addition to international standard Good Clinical Practice (GCP) and vaccine trial requirements, COVID-19 studies also require additional considerations on safe-distancing within site facilities, appropriate Personal Protective Equipment (PPE), and specialized laboratory equipment for both investigational product storage and SARS-CoV-2 diagnostics [14]. Supporting vaccine studies in LMICs during this time was paramount to reducing global disparities within the COVID-19 vaccine development landscape.

With this need in mind, the Gates Foundation (GF) launched an initiative to rapidly scale-up clinical trial readiness of sites in Asia, Africa, and Latin America through product development partners (PDPs). The Instituto D’Or de Pesquisa e Ensino (IDOR; Rio de Janerio, Brazil) was the primary PDP working with sites in Latin America [15] while the International Vaccine Institute (IVI; Seoul, South Korea) and the Program for Appropriate Technology in Health (PATH; Seattle, USA) worked with sites across Asia and Africa. This article describes the site readiness project carried out by the IVI and provides an overview of the site selection process, critical components of capacity building, and final assessment of site capacity.

## METHODS

The objective of the project was to enable study sites in LMICs to conduct large-scale SARS-CoV-2 vaccine efficacy trials with rapid enrollment—ideally enrolling 800 to 1000 participants per month at each site—while maintaining rigorous ethics and research quality. As a PDP, the IVI was awarded the GF grant (INV-021581) to support trial sites in LMICs through targeted capacity advancement and preparation activities. The grant funds were expected to train staff, improve infrastructure, and procure essential equipment in order to facilitate a highly qualified site ready to conduct a vaccine trial within four weeks of approvals.

**Figure 1.**
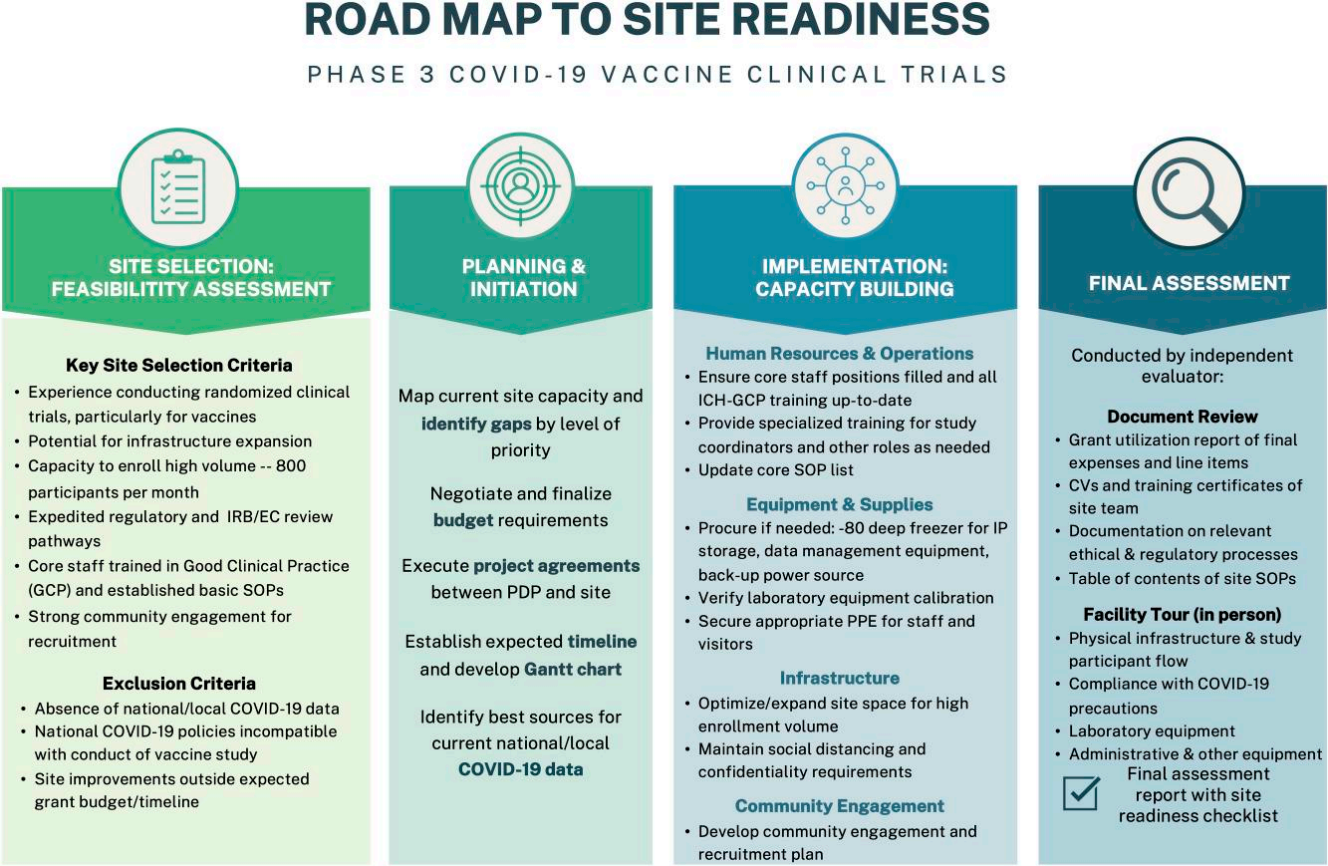
Site readiness project road map. Abbreviations: COVID-19, coronavirus disease 2019; IRB, institutional review board; EC, ethical committee; SOP, standard operating procedure; IP, investigational product; PPE, personal protective equipment; CV, *curriculum vitae*.

This capacity enhancement project followed a common road map across all sites: site selection, planning and initiation, implementation, and final evaluation. A brief summary of each stage of the project road map can be seen in Figure 1.

### Selection of Sites

Site selection was based on the site's previous experience and existing human resources, laboratory capacity, study operations, physical infrastructure, and community engagement. Characteristics of the site country were also taken into account, including the country's regulatory environment, COVID-19 data availability, and relevant COVID-19 public health policies. A standardized feasibility form was developed to make a preliminary assessment of these site and country features indicating potential to conduct large-scale vaccine trials.

Potential sites were identified based on the IVI's previous study collaborations, existing relationships, and institutional network. After an initial introduction to the capacity enhancement objectives and expected timeline, the sites completed the feasibility form within one week. Follow-up meetings were held if any questions or clarifications were needed to make final site selection decisions.

The site selection criteria are included in Figure 1. Not having one of these features was not disqualifying, and sites were assessed holistically. For example, sites that lacked experience conducting vaccine trials but demonstrated proficiency in other clinical trials were still considered.

Sites could be excluded from consideration if the site: (1) was in a country or locality without up-to-date COVID-19 epidemiological data; (2) was in a country with COVID-19 policies that would preclude conduct of a vaccine trial; or (3) the cost of qualifying the site was beyond the scope of the grant or readiness timeline.

The IVI team used the above criteria to assess sites for their potential to carry out rapid preparations and capacity building. Given the project aim that designated trial sites should be operational to conduct a Phase 3 vaccine trial as early as 4 months from selection into the readiness project, the sites’ ability to work within expected timelines was prioritized over other factors.

### Planning and Initiation

Selected sites moved forward with the planning and formal initiation stages of the project. Building upon the earlier feasibility assessment, the IVI and site teams conducted a comprehensive mapping of current site capacity by reviewing equipment and supply inventories, laboratory capabilities, study team experience, approved Standard Oeprating Procedures (SOPs), staff training logs, site building blueprints, and work station flow charts. With this baseline assessment, potential gaps were identified based on the anticipated demands of high enrollment volume, COVID-19 prevention guidelines, COVID-19 candidate vaccine storage requirements, additional staffing needs, and more.

Site budgets were developed primarily by site teams, with feedback and negotiation from the IVI team. Wherever possible, current quotations for equipment costs were received from local vendors; in cases where exact quotations were not possible or overly delayed, past purchase costs were used. Formal agreement between the IVI and each site were executed once site budgets were finalized.

To ensure smooth and timely project delivery between partners, a basic communication plan was developed establishing regular meeting times, update frequency, and critical points-of-contact. A Gantt chart with key milestones was used for progress tracking and presenting timeline expectations.

In addition, the constantly evolving pandemic conditions necessitated regular monitoring of the site country's COVID-19 context, which would affect the site's suitability for a vaccine trial. The site teams identified sources for the most up-to-date reports on COVID-19 cases, hospitalizations, and epidemiological data at the country and local-levels. Any reports of updated COVID-19 policies within the country were also monitored.

## IMPLEMENTATION OF CAPACITY ADVANCEMENT

### Human Resources & Operations

Training was tailored to the needs of each site's previous experience. Virtual ICH-GCP training was provided through the Collaborative Institutional Training Initiative (CITI) program, The Global Health Network (TGHN), and other certified training organizations. In-person training and workshops by the IVI's clinical operation team were provided to sites with less experience in Phase 3 clinical trials. Study coordinators underwent further training and assessments to demonstrate competency. Less experienced coordinators were asked to complete an online course called “Open Access Clinical Research Operations for Study Coordination,” hosted by the Faculty of Capacity Development. The core skills and experience of the more seasoned coordinators were evaluated using “The TGHN's Competency Framework” [16]. Upon successful completion of training, study coordinators shared certifications of completion or competency scoring.

All core staff were verified as meeting basic qualification requirements (e.g., current medical licensing; good standing with their respective national regulatory authorities) to conduct research and having role-based training in Good Clinical Practice (GCP), Good Laboratory Practice (GLP), Human Subjects Protections (HSP), and Responsible Conduct of Research (RCR).

The IVI and site teams jointly determined the staffing needs for conducting a high-volume vaccine trial, with further classification for “essential” positions. Essential positions were the core study team that were expected to be fully on-staff presently. Remaining positions were those who could be quickly hired and onboarded in the event of a clinical trial agreement. Given the high staffing needs to carry out a rapid enrollment strategy, the non-core staff positions may not be fully retained without an active study due to budgetary concerns. Instead, a roster of qualified and available hiring candidates was developed.

The teams developed a list of essential SOPs, including those on COVID-19 prevention measures, and worked to fill any gaps in the current SOP suite. All updated and newly developed SOPs were reviewed and approved.

### Equipment and Supplies

Essential materials for conducting COVID-19 vaccine trials were procured. Specific storage requirements of mRNA vaccine candidates called for the availability of −70°C and below ultra-low temperature freezers. In the laboratory, the teams ensured that basic equipment for processing large sample volumes such as centrifuges and biosafety hoods were in place. Validation and calibration certificates for all laboratory equipment were verified. Sites established a reliable supply of appropriate PPE for both staff and participants such as face mask, gloves, isolation gowns, and eye protection include cleaning and hygiene kits, i.e., soap, hand sanitizer, and cleaning supplies. Essential clinical trial materials were also procured if necessary. These included back-up power source, medical emergency management supplies, and chairs/furnishing for participants, and laptops.

### Infrastructure

Site infrastructure included all areas related to study workstations such as screening, randomization, blinding, monitoring, investigational product (IP) administration and storage, documentation, and medical emergency management. Site infrastructure was optimized for the specific needs of COVID-19 studies. These included: adequate space for social distancing, outdoor waiting areas where possible, and separated areas for management of COVID-19-positive study participants.

Infrastructure improvements were also made in undertaking the goal of enrolling 800 to 1000 participants per month. Additional areas were needed to increase the number of work stations for medical intake, informed consent, and specimen collection. Sites used a combination of constructing booths, using room dividers, and improving previously vacant buildings to achieve these needs. In some cases, satellite sites were included in the strategy for increasing recruitment and enrollment volume. Ensuring participant confidentiality and following COVID-19 prevention measures were the highest priorities in planning these additional areas.

### Community Engagement

Community engagement plans were reviewed and past experiences with study recruitment were discussed. The site teams considered how the subject of COVID-19 vaccines necessitated added sensitivity and communication, due to circulating vaccine misinformation in many communities [17, 18]. With the goal of rapid recruitment for a future vaccine study in mind, strategies to bolster community engagement and expand recruitment efforts were developed.

Ethics and regulatory authorities were also engaged to secure a clear understanding of expedited review pathways and expectations for a COVID-19 vaccine trial. The ever-evolving pandemic situation required close attention to how national and institutional authorities would approach a COVID-19 vaccine trial.

## FINAL ASSESSMENT

A final site assessment was conducted by an independent evaluator to validate capacity advancement activities and provide an unbiased evaluation of the site's readiness to implement large-scale COVID-19 vaccine efficacy trials. Qualified consultants with clinical research operation backgrounds and no affiliation with the site, the IVI, or the GF carried out the final assessments.

The independent evaluators were appointed to evaluate sites on not only standard clinical trial readiness benchmarks, such as staff training and laboratory equipment, but also COVID-19-specific requirements. The IVI team provided the independent evaluator with a site readiness checklist form, which was developed with feedback from the GF grant team and the other PDPs—PATH and IDOR—at the start of the project. The consultants were also briefed on the site's baseline feasibility data and the main capacity building activities conducted for each site.

The assessment process involved a document review segment that was largely conducted through virtual meetings and communications, and a facility tour segment with at least one in-person site visit by the evaluator. For document review, site teams were asked to provide the (1) grant utilization report, specifying the activities carried out and the expenses incurred, (2) training certificates and CVs of the investigator and staff, (3) documentation on ethical and regulatory processes, and (4) table of contents for site SOPs. For the facility tour, the evaluator assessed the site's physical infrastructure, compliance with COVID-19 precautions, and clinical and laboratory equipment. During the visit, the site investigator and other relevant staff were also interviewed to gather qualitative insights on site readiness.

Once the evaluation was complete, the independent consultant shared the final assessment report for each site with the IVI team. The report detailed the reviewed documents and site facilities, the site readiness checklist completed by the consultant, any remaining capacity gaps or recommendations, and the overall outcome of the assessment.

## RESULTS

The site readiness project was initiated in September 2020 and was completed by May 2021. The final site selection was confirmed by the end of October and was immediately followed by initiating site preparedness activities. All sites completed essential capacity advancement and necessary infrastructure preparedness by April 2021. Final assessments were concluded in May 2021.

### Site Selection

**Figure 2.**
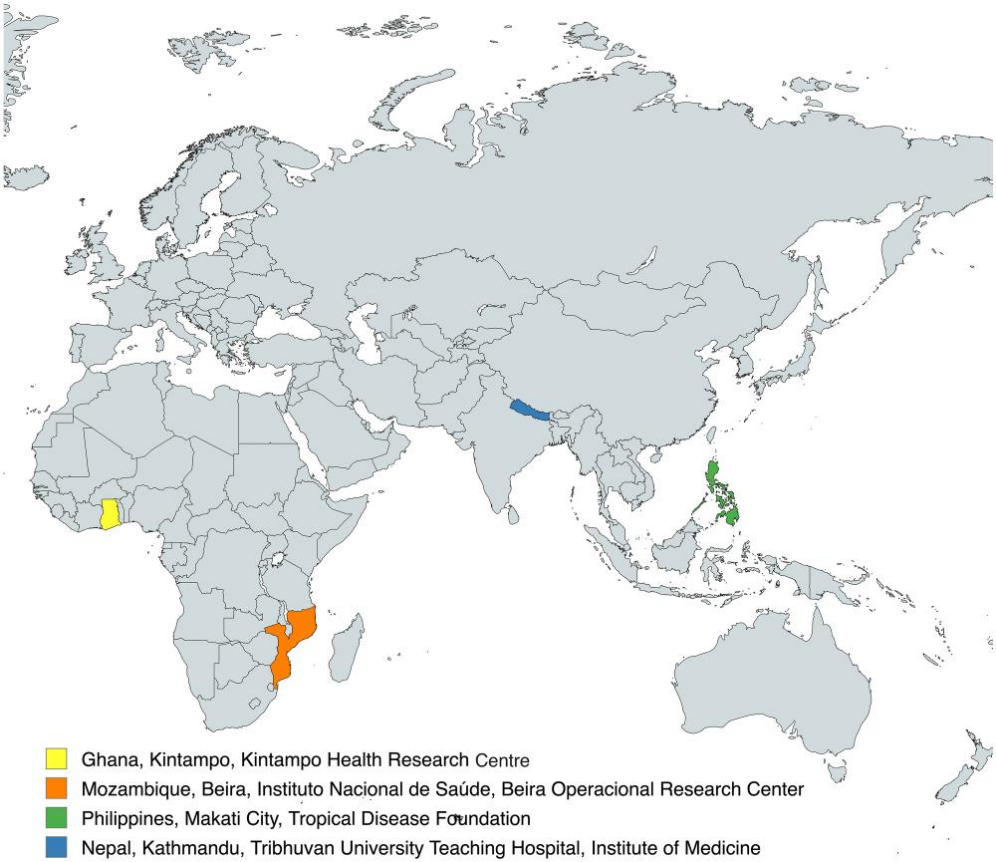
Selected sites.

The IVI team engaged six sites in Ghana, Mozambique, Burkina Faso, Nepal, the Philippines, and Indonesia. After early discussions and feasibility assessments, two sites opted out of the selection process: the Burkina Faso site based on conflicts with existing study commitments and timelines, and the Indonesia site based on the country's emergency use authorization of the Sinovac vaccine that was incompatible with vaccine trials [19]. The four sites based in Ghana, Mozambique, Nepal, and the Philippines were included in the final site readiness initiative and are listed in Figure 2.

### Baseline Characteristics

All four selected sites had experience with conducting randomized clinical trials; however, two of the site principal investigators did not have experience with a Phase 2 or 3 vaccine clinical trial. Roughly 80% of core staff across all four sites had previous training in ICH-GCP, and some lacked specialized role-based training. All sites had either additional building space or available satellite sites for expansion. At baseline, all of the sites had gaps in equipment that would be necessary to handle an mRNA COVID-19 vaccine trial (eg, no sites were equipped with deep freezers with the enough capacity to store clinical trial IP, some sites lacked adequate centrifuges and other equipment for processing high volumes of specimen). A brief summary of baseline characteristics by site are listed in Table 1.

**Table 1.** Baseline Site Characteristics

	Kintampo Health Research Centre (Kintampo, Ghana)	Instituto Nacional de Saúde, Beira Operacional Research Center (Beira, Mozambique)	Tropical Disease Foundation (Makati City, Philippines)	Tribhuvan University Teaching Hospital, Institute of Medicine (Kathmandu, Nepal)
Experience in conducting clinical trials	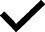	Not in vaccine clinical trials	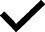	Not in vaccine clinical trials
Potential for infrastructure expansion	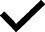	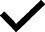	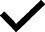	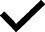
Expedited regulatory and IRB/EC review pathways	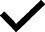	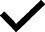	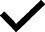	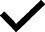
Core staff qualified and trained in GCP	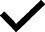	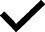	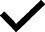	In-depth GCP training requested
Strong community engagement	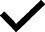	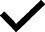	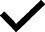	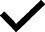
Capacity to enroll high volume of participants	Gaps: insufficient number of study work stations; insufficient physical space for social-distancing; lack of separate area for management of COVID-19 positive participants			
Laboratory capacity for COVID-19 study	Gaps: lack of ultra-low temperature freezers for IP storage; inadequate specimen processing equipment capacity			
National and regional COVID-19 epidemiological data available	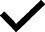	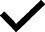	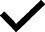	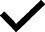

Abbreviations: COVID-19, coronavirus disease 2019; EC, Ethics Committee; GCP, Good Clinical Practice; IRB, Institutional Review Board.

### Capacity Advancement Grant Utilization

**Figure 3.**
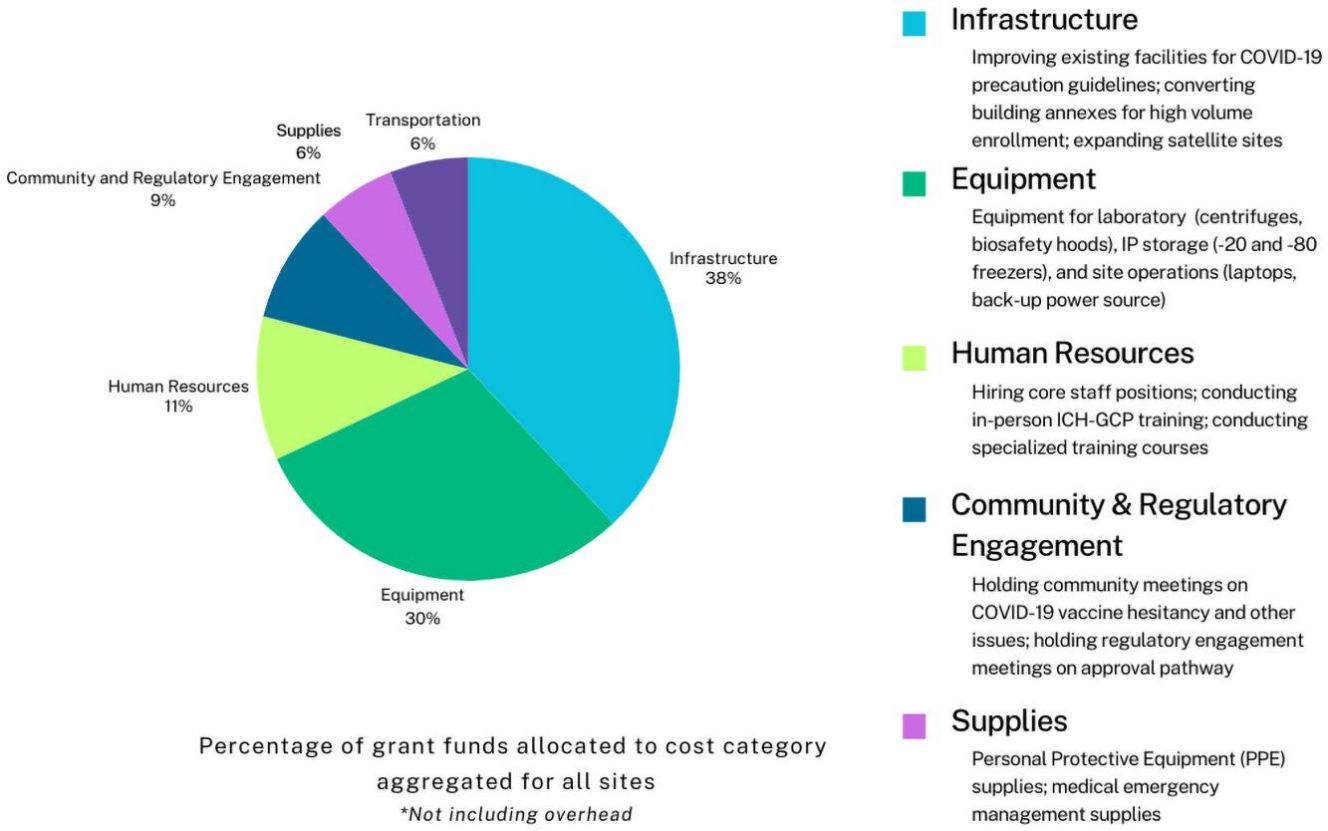
Grant utilization by category.

Most funds were oriented toward purchasing site and laboratory equipment (such as laboratory-grade freezers, fridges, and centrifuges) and administrative materials. Funds were also dedicated to constructing or renovating physical space, hiring staff, delivering training sessions, monitoring epidemiological data, and site security. Across all four sites, 32% of grant funds were allocated to the expansion and renovation of facilities; 29% to purchasing site equipment; 11% to personnel; 6% to transportation; 5% to procuring supplies; and 9% to community and regulatory engagement; as shown in Figure 3.

### Challenges

The conditions of the ongoing COVID-19 pandemic posed the greatest overall challenge to the site preparation activities. The worldwide demand for equipment and consumables created significant procurement delays in all sites [20]. Equipment (such as freezers, centrifuges, and laptops) and PPE supplies were priced at many multiples of normal market value and experienced longer-than-normal shipment times. Materials for infrastructure improvements were also costlier than expected, or not delivered on time. Site personnel and the IVI team were in constant communication to identify alternative distributors, negotiate costs, and monitor shipment timelines.

Various other external factors outside of the teams control affected the project progression. In January 2021, tropical cyclone Eloise made landfall in Beira City, Mozambique, and caused devastating damage in the community and stalled activities at the Instituto Nacional de Saúde, Beira-Mozambique site [21]. In all four countries, intermittent lock-down measures created challenges to planned site trainings and activities.

### Final Assessment

Independent evaluators conducted final assessments with both virtual and in-person visits as planned. Their observations concluded that all sites were able to meet the specific requirements of a COVID-19 study, and were ready to conduct a Phase 3 pivotal efficacy trial. By the conclusion of this site readiness project in June and July 2021, all sites were in discussions with trial sponsors to conduct COVID-19 studies [22, 23].

## DISCUSSION

The GF-supported readiness initiative described in this paper was able to prepare LMIC sites for pivotal Phase 3 COVID-19 vaccine trials while also investing in the longer-term capacity for health research in these countries. Under this initiative, four sites were successfully established across Asia and Africa in under seven months, and all sites went on to conduct impactful COVID-19 studies.

Sites were selected for inclusion into the initiative with key considerations for existing capacity, readiness, and ability to conduct high quality, GCP-compliant study trial. The site readiness objective was for sites to be capable of conducting a COVID-19 vaccine trial with high-volume enrollment strategy within 4 months of the project. Capacity advancement activities were tailored to support the unique needs of each site. Despite facing the challenges of carrying out these activities through pandemic-related lockdowns, supply chain delays, and natural disasters, the initiative successfully fulfilled all key site readiness expectations. The final assessment, conducted by an independent evaluator, found that all sites were ready and capable of conducting a large-scale COVID-19 vaccine efficacy trial with qualified staff, operations, equipment, and infrastructure.

In conclusion, the disparities in vaccine clinical trials faced by LMICs represent urgent issues that require immediate global attention. The evident need for more sustainable research capacity enhancement and the establishment of adequate pandemic preparedness infrastructures in LMICs has resulted in growing efforts to those ends. Large donors are playing significant roles in funding and overseeing such essential improvements in Africa. For instance, the Advancing Research Capacity in West Africa (ARC-WA) project is an initiative supported by CEPI to strengthen clinical trial sites across West Africa [24]. With the IVI and the Medical Research Council Unit, The Gambia (MRCG) at the London School of Hygiene & Tropical Medicine (LSHTM) as technical coordinating partners, the ARC-WA conducts a systematic site assessment and subsequent selection for targeted capacity strengthening for 25–30 clinical trial sites in the coming 2–3 years. The Africa CDC, along with the study sites, is the owner of this activity to improve research preparedness in regions of Africa.

The ARC-WA initiative is currently being implemented through two interrelated streams of work: (1) capacity strengthening of clinical trial sites in West Africa to support Phase 2b and 3 clinical trials; and (2) a broader agenda for emergency evidence generation, working with governments, coordination platforms, regulators, and the broader ecosystem. Maintaining active clinical research sites and researchers is crucial for testing systems, honing skills, and retaining talent in advance of any future pandemic. Overall, ARC-WA seeks to enable countries in West Africa and institutional partners, particularly the Africa CDC, to respond more quickly to outbreaks through rapid vaccine development and increased collaboration between partner countries. Developing similar infrastructure in Asian countries where it does not yet exist would also be important for addressing global health risks.

Finally, three major pillars are critical to leveraging the current capacity strengthening momentum. First, the Africa CDC is responsible for coordinating pan-African initiatives, such as those described in this paper, on pandemic preparedness, surveillance, outbreak detection, diagnostics, and, eventually, the development of vaccines. Second, responsibilities should be transferred to study sites and in-country leadership; this is high on the agenda of many programs, including the ARC-WA project. Third, longer-term strategic partnerships and sustainable investment into health research capacity building must be secured. All of the above will achieve a more resilient global health system that is far more capable of dealing with future pandemics and protecting people. Thus, this article calls for stronger partnerships and encourages collaboration between leading investors, governments, scientific organizations, and communities. By combining resources, expertise, and influence, we can drive significant advancements and address global health challenges more effectively.

## Notes

***Author contributions.*** All authors met the International Committee of Medical Journal Editors (ICMJE) criteria for authorship and were responsible for the conception and conduct of this site readiness initiative, acquisition of data, and its analysis. All authors were involved in critically revising the manuscript for important intellectual content and approved the final manuscript.

***Acknowledgments.*** The authors of this article express gratitude to the Gates Foundation and CEPI, the funders of this ground-breaking and strategic project for capacity building in global health. Furthermore, we extend appreciation to the investigators and staff of the clinical sites for their dedicated participation despite the project's limited timeline.

***Data sharing statement.*** The Gates Foundation group has regularly shared the project data via the COVAX website—Dashboard of COVAX-Supported Sites Ready for Clinical Trials (https://epi.tghn.org/covax-overview/clinical-science/clinical/#ref.

***Financial support.*** This work was supported by the Gates Foundation, Seattle, Washington, USA (grant numbers INV-021581 and INV-021539) and the Coalition for Epidemic Preparedness Innovations (CEPI).

***Supplement sponsorship.*** This article appears as part of the supplement “COVID-19 Vaccine Research in Africa,” sponsored by the International Vaccine Institute and funded by the Coalition for Epidemic Preparedness Innovations (CEPI).
